# Differences in personality functioning impairment in mood, anxiety, and personality disorders: a cluster analysis

**DOI:** 10.1186/s12888-022-03958-4

**Published:** 2022-05-04

**Authors:** Nikola Doubková, Radek Heissler, Marek Preiss, Edel Sanders

**Affiliations:** 1 National Institute of Mental Health, Topolová 748, 250 67 Klecany, Czech Republic; 2 Faculty of Education, Charles University, Prague, Czech Republic; 3 University of New York in Prague, Prague, Czech Republic

**Keywords:** personality functioning, personality disorders, mood disorders, anxiety disorders

## Abstract

**Background:**

The Alternative DSM-5 Model for Personality Disorders and the latest eleventh version of the International Classification of Diseases implement the level of impairment in self and interpersonal personality functioning (Level of Personality Functioning Scale - LPFS) as a core feature of personality pathology. However, some studies have indicated that personality functioning is also impaired in other mental disorders, but a more thorough exploration is missing. Thus, this study aims to develop profiles of levels of personality functioning in people with personality disorders and some other psychiatric diagnoses as well as without diagnosis.

**Methods:**

One-hundred-forty-nine people participated in the study. They came from three groups – healthy controls (*n* = 53), people with personality disorders (*n* = 58), and people with mood and anxiety disorders (*n* = 38). The LPFS was assessed by the Semi-structured Interview for Personality Functioning DSM-5 (STiP-5.1). An optimal clustering solution using agglomerative hierarchical cluster analysis was generated to represent profiles of personality functioning.

**Results:**

The two patient groups showed significantly higher levels of personality functioning impairment than healthy controls. People with personality disorders showed higher levels of impairment than the other groups. In addition, the clustering analysis revealed three distinct profiles of personality functioning.

**Conclusions:**

The impairment of personality functioning seems to be useful in the clinical assessment of other than personality disorders as well. As the resulting clustering profiles suggest, LPFS can be seen as an overall indicator of the severity of mental health difficulties and the presence of mental disorders symptoms. The LPFS provides valuable and detailed information about the individual’s mental health and can thus serve as a broad basis for case formulation, treatment and therapy planning, and prognosis.

## Background

The Level of Personality Functioning Scale (LPFS) was introduced in the fifth edition of the Diagnostic and Statistical Manual of Mental Disorders (DSM-5) [1] as Criterion A of the Alternative Model for Personality Disorders (AMPD), which is a dimensional-categorical hybrid. The AMPD defines personality functioning as a dimensional construct disturbed on a continuum. Therefore, the level of personality functioning is assessed from 0 (little or no impairment) to 4 (extreme impairment). At least moderate impairment (level 2) is required for the diagnosis of a personality disorder. The global level of personality functioning is assessed in two domains: Self and Interpersonal functioning. Self-functioning comprises the two elements Identity and Self-direction, while Interpersonal functioning consists of Empathy and Intimacy elements. The concept of LPFS is transtheoretical, informed by divergent theoretical and conceptual approaches [2–4].

The eleventh edition of the International Classification of Diseases (ICD-11) [5] includes a similar but fully dimensional approach to personality disorders diagnosis. In this model, the central manifestations of personality disorders are impairments in functioning of aspects of the self (e.g., identity, self-worth, self-direction) and/or problems in interpersonal functioning (e.g., developing and maintaining close and mutually satisfying relationships, understanding others’ perspectives, managing conflicts) [5].

The traditional categorical model of personality disorders and psychopathology, in general, was found to have many shortcomings and problems [6–8]. The dimensional models of personality disorders were introduced to address these limitations and enhance clinical utility. However, the notion of dimensionality of personality pathology is not new, although it has come into focus just recently [9]. Indeed, personality is an “umbrella organization” [10] including several components, in which healthy development could be disturbed in many ways even in people not experiencing mental health issues, as it is currently concluded that psychopathology exists on a continuum with normal-range functioning [11]. One of the advantages of the dimensional models is the gain of a more detailed clinical characterization, with specifications about the level of impairment in different domains, which allows for more accurate and individualized treatment and therapeutic planning or case formulation [1, 12–14]. Moreover, it gives researchers a framework for facilitating more ecologically valid studies leading to evidence-based psychiatric and psychological intervention targeting [6].

The general severity criterion of personality disorders in the AMPD and ICD-11 are congruent and share common characteristics [15]. To this day, instruments operationalizing ICD-11 levels of severity have been scarce and no clinician-rated interview has been introduced so far. Thus, instruments assessing Criterion A have been used in studies and obtained valid results [15–18]. In the assessment of personality disorders, the use of semi-structured interviews particularly is recommended [19], e.g., the Semi-structured Interview for Personality Functioning DSM-5 (STiP-5.1) [20] was found to be valid and an easy-to-use tool applicable for both AMPD and ICD-11 [15].

The personality functioning was conceptualized to assess impairment and delayed development of the adaptive intrapsychic system needed for mature fulfillment in adult life [12, 21]. Thus, it is plausible to assume that personality functioning is to some degree also impaired in other mental disorders in addition to personality disorders [4, 22–26]. Furthermore, the interest in personality functioning in other mental disorders than personality disorders has increased in recent years [25].

It was suggested that various mental disorders could be associated not only with the different global severity level of personality functioning impairment but apparently with different patterns of impairment in the self and interpersonal domains as well [17, 21, 22, 25]. For example, Di Pierro et al. [24] found that people with psychopathologies other than personality disorders showed impairment in facets of personality functioning (i.e., identity, empathy), however, the impairment just rarely satisfied the Criterion A of the AMPD. Møller et al. [26] found associations between impairments in personality functioning and posttraumatic stress disorder, with possible further utilization in the differential diagnosis. There are also initial results indicating that personality functioning can have an impact on psychosocial functioning across various diagnostic categories [27]. Though the utility of personality functioning assessment beyond personality disorders was repeatedly supported by research [17, 22, 24, 26, 27], with some authors even proposing that personality functioning might represent a general psychopathology factor [28–30], little is still known about the nature of personality functioning impairment in other-than-personality-disorders diagnoses.

Therefore, using cluster analysis, this study aims to develop profiles of levels of personality functioning in people with personality disorders and other psychiatric diagnoses as well as without diagnosis. We are interested in (1) differences in personality functioning disturbances between groups of people with various mental disorders (e.g., personality disorders with or without comorbidity, mood and/or anxiety disorders) and people without diagnosis, and (2) exploring whether these mental disorders show different patterns of associations with not only global personality functioning but also its domains – self and interpersonal – and facets. The goal is to delineate groups based on their personality functioning, assessed by the STiP-5.1 [20], further describe characteristics of each group, especially related to symptoms of anxiety and depression, and evaluate their clinical meaningfulness as well as the possibility of interpretation and implementation in clinical practice for assessment of personality functioning.

## Methods

### Study design

Ethical approval for this study was obtained from the local ethics committee of the National Institute of Mental Health (approval number 107/18) on March 28, 2018. All participants were informed about the goals and procedures of the study, and all participants signed written informed consent before participating in the study. Data for this study were collected between January 2019 and October 2020. Two psychiatric patient groups were recruited at the inpatient ward and the daycare center of the National Institute of Mental Health, Czech Republic. The group of healthy control subjects was recruited via leaflets, advertising, and the snowball sampling technique. All participants received a monetary reward for their participation of approx. € 25 (600 CZK).

Patients were diagnosed according to ICD-10 [31] by their attending psychiatrist and psychologist through standardized diagnostic interviews and a battery of tests during a therapeutic program; presented here are their discharge diagnoses. All participants underwent the Semi-structured Interview for Personality Functioning DSM-5 [20]. All interviewers were psychologists and underwent basic training led by Joost Hutsebaut, an author of the interview. All interviewers met at several consensus meetings to maintain their rating consistency. After the interview, participants filled in questionnaires.

Dolnicar et al. [32] recommend that the optimal sample size for cluster analysis should range between 30 to 70 times the number of variables, while 70 represents the most conservative requirement. In this study, we included three variables in the cluster analysis; therefore, our sample size (*N* = 149) roughly corresponds to 50 times the number of variables and should be adequate.

### Participants

In total, 149 participants were included in the study. Demographic characteristics are given in Table 1. Diagnoses according to ICD-10 [31] are given in Table 2. Inclusion criteria for all subjects were: 1) age ≥18 years, 2) Czech citizenship. Exclusion criteria for all participants were: 1) organic brain disease, 2) cognitive impairment.

**Table 1:** Sample characteristics (*N* = 149)

	Controls (*n* = 53)	Anxiety and mood disorder (*n* = 38)	Personality disorder (*n* = 58)
	Mean (SD)	Mean (SD)	Mean (SD)
**Age (years)**	33.8 (12.8)	37.9 (13.5)	31.5 (9.98)
**Gender**			
	n (%)	n (%)	n (%)
**Female**	31 (58.49)	23 (60.53)	42 (72.41)
**Male**	22 (41.51)	15 (39.47)	16 (27.59)
**Family status**			
**Single**	35 (66.04)	22 (57.89)	45 (77.59)
**Married**	9 (16.98)	10 (26.32)	5 (8.62)
**Divorced**	9 (16.98)	6 (15.79)	7 (12.07)
**Widowed**	0 (0.0)	0 (0.0)	1 (1.72)
**Education**			
**Primary level**	0 (0.0)	5 (13.16)	13 (22.41)
**Secondary level**	26 (49.06)	19 (50.0)	35 (60.35)
** Tertiary level**	27 (50.94)	14 (36.84)	10 (17.24)

**Table 2:** ICD-10 diagnoses (*N* = 149)

	Anxiety and mood disorders group (*n* = 38)	Personality disorders group (*n* = 58)
**Bipolar affective disorder (F31.2, F31.3, F31.4)**	7 (18.4%)	-
**Depressive episode (F32.0, F32.1, F32.2)**	2 (5.3%)	-
**Recurrent depressive disorder (F33.0, F33.1, F33.2)**	8 (21.1%)	-
**Phobic anxiety disorders (F40.0, F40.1, F40.8)**	3 (7.9%)	-
*in comorbidity with F41.3*	*1 (2.6%)*	
**Other anxiety disorders (F41.0, F41.1, F41.2, F41.3)**	6 (15.8%)	-
*in comorbidity with F45.1, F32.0*	*2 (5.3%)*	
**Obsessive-compulsive disorder (F42.0, F42.1, F42.2)**	8 (21.1%)	-
**Somatoform disorders (F45.1, F45.9)**	1 (2.6%)	-
**Dissocial personality disorder (F60.2)**	-	3 (5.2%)
**Emotionally unstable personality disorder (F60.3)**	-	30 (51.7%)
*in comorbidity with F31.4, F33.0, F33.1, F40.0, F41.2, F42.2*		*6 (10.3%)*
**Histrionic personality disorder (F60.4)**	-	2 (3.4%)
*in comorbidity with F43.2*		*1 (1.7%)*
**Anankastic personality disorder (F60.5)**	-	1 (1.7%)
**Anxious personality disorder (F60.6)**	-	1 (1.7%)
*in comorbidity with F42.2*		*1 (1.7%)*
**Dependent personality disorder (F60.7)**	-	1 (1.7%)
**Other specific personality disorders (F60.8)**	-	4 (6.9%)
*in comorbidity with F32.2, F42.2*		*2 (3.4%)*
**Mixed and other personality disorders (F61)**	-	3 (5.2%)
*in comorbidity with F32.1, F40.0, F41.2*		*3 (5.2%)*

*Note*. Stated are diagnostic categories and codes according to 10^th^ edition of International Classification of Disease (ICD-10).

In our study, we included three groups of participants. The patients’ groups were further divided into subgroups for some analyses.

1. Healthy control subjects (*n* = 53, of which 31 were females). Additional exclusion criteria were implemented: 1) presence of depression symptoms (score >20 on Beck Depression Inventory [33]) or anxiety symptoms (score >18 on Beck Anxiety Inventory [34]), 2) current or previous psychiatric treatment or hospitalization.
2. Patients with anxiety and mood disorders (*n* = 38, of which 23 were females) were recruited from a group of adults seeking psychological treatment at a local mental health hospital. Inclusion criteria were: 1) current or previous psychiatric treatment or hospitalization, 2) having a diagnosis of mood disorders (F30-F39 according to ICD-10) or anxiety, stress-related, and somatoform disorders (F40-F48 according to ICD-10) given by an attending psychiatrist. Overall, 17 (44.74%) participants had mood disorder diagnosed, and 21 (55.26%) had anxiety disorder diagnosed (for details see Table 2). We formed two subgroups from these participants: 2a) participants with mood disorders (*n* = 17; 9 females), and 2b) participants with anxiety disorders (*n* = 21 of which 14 were females). Only 3 participants (7.89%) had two diagnoses, all were included in the anxiety disorders subgroup because it was indicated as their primary diagnosis.
3. Patients with personality disorders (PD) (*n* = 58, 42 females) were also recruited from a group of adults seeking psychological treatment at a local mental health hospital. Inclusion criteria were: 1) current or previous psychiatric treatment or hospitalization, 2) having a personality disorders diagnosis (F60, F61 according to ICD-10) given by an attending psychiatrist. These participants, due to reasons given by Doering et al. [22], were further divided into two subgroups based on the present comorbidity: 3a) participants with a personality disorder comorbid with mood or anxiety disorder (*n* = 13, 8 females), 3b) patients with a personality disorder without comorbidity (45 of which 34 were females). Detailed information about psychiatric classification and comorbidity are given in Table 2.

### Measures

#### Demographic data

A short demographic questionnaire assessed age, gender, family status, education level, and psychiatric treatment/hospitalization experiences.

#### The Semi-structured Interview for Personality Functioning DSM-5 (STiP-5.1)

The Semi-structured Interview for Personality Functioning DSM-5 (STiP-5.1) [20] is a semi-structured clinician-rated interview assessing the global level of personality functioning according to the Alternative Model of Personality Disorders, introduced in Section III of the DSM-5 [1], and now also used for the assessment of personality functioning according to ICD-11 [15]. The interview consists of 28 open questions and optional clarifying questions. The interview is divided into 12 indicators or facets, which is the label used by the authors of STiP-5.1, (i.e., uniqueness, self-esteem, emotions, goals, standards, self-reflection, understanding others, perspectives, impact, relationships, closeness, mutual respect); each of them is rated on five levels of severity ranging from little or no impairment (0) to extreme (4) impairment. The facets construct elements of Identity, Self-direction, Empathy, and Intimacy, combined in the domains of Self (Identity and Self-direction) and Interpersonal functioning (Empathy and Intimacy). The interviewer aggregates the total score based upon the evaluation of facets scores. STiP-5.1 shows very good psychometric properties [20, 35, 36]. Due to organizational reasons, we were not able to assess interrater reliability (ICC), however, previous studies using this interview showed good to excellent ICCs even after modest administration training and in various study samples [15, 20, 36–38]. Internal consistency, measured by McDonald’s ω, was high in our sample (total = 0.936, Self = 0.898, Interpersonal = 0.89). In our study, in addition to the total severity score, we use the two domain scores – Self and Interpersonal functioning – and twelve facets scores as well (as already done by previous studies exploring personality functioning [26, 27]).

#### Beck Depression Inventory (BDI)

Beck Depression Inventory (BDI) [33] is a brief 21-item self-report inventory used to assess the severity of depressive symptoms. Higher scores indicate a higher prevalence of depressive symptoms. In our sample, the internal consistency of BDI measured by McDonald’s ω was 0.957.

#### Beck Anxiety Inventory (BAI)

Beck Anxiety Inventory (BAI) [34] is a brief 21-item self-report inventory used to assess the severity of anxiety symptoms. Higher scores show a higher prevalence of anxiety symptoms. The internal consistency of BAI in our sample, measured by McDonald’s ω was 0.926.

### Data analysis

Data was analyzed in Rstudio (version 1.4.1106) using the following libraries: cluster [39], ggpubr [40], gmodels [41], fpc [42], rstatix [43]. The Shapiro-Wilk test for normality showed that scores are not normally distributed (W ranging from 0.829 to 0.948, *p* < 0.001). Additionally, Fligner-Kileen’s test showed that groups’ variances in most of the scales are significantly different and significantly inhomogeneous. Therefore, non-parametric tests were used. The Kruskal-Wallis H test (effect size given by η^2^ with values < 0.01 interpreted as small, and above 0.14 as large effect sizes [44]) was used for group comparisons of the level of personality functioning, BDI, and BAI scores. Dunn’s post-hoc test of multiple comparisons was conducted with Bonferroni’s correction. Categorical variables were compared using Fisher’s exact test.

We applied hierarchical agglomerative clustering with Ward’s linkage using Euclidean distance [45]. No demographic variables appeared to have a systematic influence on personality functioning (apart from the level of education, yet it seems to reflect a more general trend and consequence of disorder related obstructions, e.g., [46, 47]), thus only the STiP total severity score together with the Self and Interpersonal domain scores were included in the cluster analysis. Since all three use the same scale, we opted not to normalize. Solutions for three to five clusters were calculated. The most suitable solution was chosen based on a combination of indices: elbow plots, dendograms, Calinski-Harabasz pseudo F-statistic, and external clustering validation (with Rand and Meila’s variation indexes) using the initial three groups as reference. Additionally, Kruskal-Wallis H test (effect size given by η^2^) was used to assess between-cluster differences in variables not included in the cluster analysis (i.e., BDI, BAI) but that we presumed to vary across clusters. The total severity score as well as two personality functioning domain scores were used for clustering, therefore analyzing differences in these three scores is rather redundant. Nonetheless, the analyses were run for all twelve facets of personality functioning to get a closer look at the nature of between-cluster differences.

## Results

### Sample characteristics

For sample characteristics, see Table 1. Males and females did not significantly differ in their age (U = 2326.5, *p* = 0.389), in family status (*p* = 0.214), nor in their level of education (*p* = 0.631). The three study groups did not significantly differ in their age (χ^2^(2) = 4.666, *p* = 0.097). The Fisher’s exact test was performed to examine whether the proportion of males and females differs between groups; the result was not significant (*p* = 0.264, two-sided). Groups also did not differ in the family status (*p* = 0.191, two-sided). However, they did differ significantly in education level (*p* < 0.001), with people with personality disorders achieving lower levels.

### Differences in the level of personality functioning

One of the goals was to find differences in personality functioning between the groups. We found significant differences between the three study groups in BAI, BDI, and levels of personality functioning (see Table 3). Post-hoc comparisons using Dunn’s test (with Bonferroni’s correction) revealed significant differences in BAI and BDI between the group of healthy controls and the groups of people with anxiety and mood disorders (*p* < 0.001), and personality disorders (PD) (*p* < 0.001); the difference between the anxiety and mood disorders group and the PD group was not significant. Differences in the STiP-5.1 overall severity score as well as two domain scores (*p* < 0.001) indicate a continuum of impairment with healthy controls having the lowest scores, i.e., showing the lowest levels of impairment, with the PD group showing scores indicating the highest levels of impairment, and the anxiety and mood disorders group being in the middle.

**Table 3:** Personality functioning and anxiety and depression scores in three study groups (*N* = 149)

	Healthy controls (*n* = 53)			Anxiety and mood disorders (*n* = 38)			Personality disorders (*n* = 58)			Kruskal-Wallis H test	
	Mean (SD)	Median	Range	Mean (SD)	Median	Range	Mean (SD)	Median	Range	χ^2^(2)	η^2^
**BAI**	5.49 (4.46)	5	0-18	20.6 (10.0)	19.5	1-44	23.2 (10.9)	23	4-52	78.387^*^	0.523
**BDI**	4.62 (4.29)	3	0-18	21.2 (10.6)	20.5	3-43	27.3 (13.2)	28.5	0-57	80.898^*^	0.54
**STiP Self**	0.19 (0.40)	0	0-1	1.29 (0.61)	1	0-2	2 (0.73)	2	1-4	99.199^*^	0.666
**STiP Interpersonal**	0.17 (0.38)	0	0-1	0.92 (0.67)	1	0-2	1.79 (0.87)	2	0-3	83.019^*^	0.555
**STiP Total**	0.13	0	0-1	1.18 (0.73)	1	0-2	2.1 (0.64)	2	1-3	103.9^*^	0.698

*Note*. * *p* < 0.001. *BAI* Beck Anxiety Inventory, *BDI* Beck Depression Inventory, *STiP* The Semi-structured Interview for Personality Functioning DSM-5

**Fig. 1:**
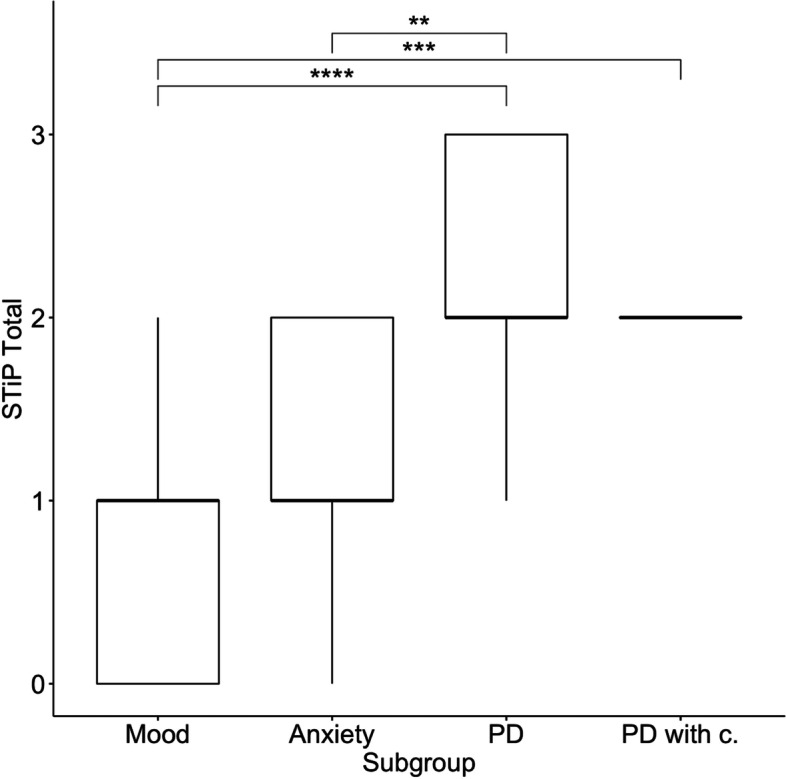
Between-subgroups differences in the STiP-5.1 total severity score (Kruskal-Wallis H test with Dunn’s post hoc test). Note. ** *p* < 0.01, *** *p* < 0.001, **** *p* < 0.0001; PD = personality disorders without comorbidity subgroup, PD with c. = personality disorders with comorbidity subgroup, STiP = The Semi-structured Interview for Personality Functioning DSM-5

We also focused on differences within the patients’ groups in personality functioning and its domains as well as anxiety and depression measures. We separated patient groups into four subgroups: 1) mood, 2) anxiety, 3) personality disorders (PD) without comorbidity, 4) PD with comorbidity. Descriptive statistics and results of this analysis are presented in Table 4. The Kruskal-Wallis H test reveals no statistically significant differences between patients’ subgroups in the BAI scores. Nonetheless, there is a statistically significant difference in the BDI score, according to Dunn’s post-hoc test, and with Bonferroni’s correction, the anxiety subgroup scores significantly lower than the PD with comorbidity subgroup (*p* < 0.01). Statistically significant differences between subgroups in personality functioning (STiP-5.1 total as well as both domain scores) were found. To explore these differences further, we applied Dunn’s post-hoc test (with Bonferroni’s correction). It turned out that the PD without comorbidity subgroup yielded significantly higher impairment in Self personality functioning than the mood disorders (*p* < 0.001) and anxiety (*p* < 0.05) subgroups as well. Moreover, the PD with comorbidity subgroup showed larger impairment in the Self domain than the mood disorders subgroup (*p* < 0.001). The subgroup of people with PD without comorbidity showed higher levels of interpersonal functioning impairment than the mood disorders subgroup (*p* < 0.001) and anxiety disorders subgroup (*p* < 0.01). Several statistically significant differences were found in the STiP-5.1 total scores (see Fig. 1). However, the mood disorders and anxiety disorders subgroups did not significantly differ in any STiP-5.1 scores, nor did the PD with comorbidity and without comorbidity groups.

**Table 4:** BAI, BDI and personality functioning in patients’ subgroups (*n* = 96)

	Anxiety and mood disorders (*n* = 38)				Personality disorders (*n* = 58)				Kruskal-Wallis H test	
	Mood (*n* = 17)		Anxiety (*n* = 21)		Without comorbidity (*n* = 45)		With comorbidity (*n* = 13)			
	Mean (SD)	Median	Mean (SD)	Median	Mean (SD)	Median	Mean (SD)	Median	χ^2^(3)	η^2^
**BAI**	17.1 (10.5)	16	23.5 (8.91)	25	24.5 (11.1)	24	18.8 (9.51)	16	8.701	0.062
**BDI**	23.1 (10.8)	23	19.6 (10.4)	19	25.3 (12.5)	26	34.1 (14.1)	37	10.493^*^	0.081
**STiP Self**	1.12 (0.7)	1	1.43 (0.51)	1	2.0 (0.77)	2	2.0 (0.58)	2	20.615^**^	0.191
**STiP Interpersonal**	0.71 (0.77)	1	1.1 (0.54)	1	1.89 (0.86)	2	1.46 (0.88)	1	24.694^**^	0.236
**STiP Total**	0.88 (0.78)	1	1.43 (0.6)	1	2.11 (0.68)	2	2.08 (0.49)	2	31.634^**^	0.311

*Note.* * *p* < 0.05, ** *p* < 0.001. *BAI* Beck Anxiety Inventory, *BDI* Beck Depression Inventory, *STiP* The Semi-structured Interview for Personality Functioning DSM-5

### Hierarchical agglomerative clustering

We used cluster analysis to examine further the differences between groups. We calculated solutions for three to five clusters. The elbow plot and dendrograms showed that k = 3 or 4 is a more suitable number of clusters in our data (Calinski-Harabasz pseudo F-statistic = 231.167 vs. 230.513, respectively). We used external clustering validation using the initial three groups as reference, which suggested that k = 3 is more suitable than k = 4 (corrected Rand index = 0.408 vs. 0.498, Meila’s variation index = 1.28 vs. 1.107, respectively). We compared the clustering result with the initial groups (Table 5). The overall accuracy of this clustering is 76.51%. This is most sensitive in detecting healthy controls (81.13%) and people with personality disorders (87.93%).

**Table 5:** Frequency table of three clusters with initial groups and subgroups as reference

Cluster	Healthy controls (*n* = 53)	Anxiety and mood disorders (*n* = 38)		Personality disorder (***n*** = 58)		Total (% of females)
		Mood (*n* = 17)	Anxiety (*n* = 21)	Without comorbidity (*n* = 45)	With comorbidity (*n* = 13)	
**1**	43	3		0		**46 (58.7)**
		3	0	0	0	
**2**	10	20		7		**37 (56.76)**
		9	11	6	1	
**3**	0	15		51		**66 (72.73)**
		5	10	39	12	

Table 6 shows the characteristics of clusters and their scores. Cluster 1 was in the mean age of 35.59 (SD = 12.72), Cluster 2 was 37.19 years old (SD = 13.99), and Cluster 3 was in the mean age of 31.02 (SD = 9.98), however, the differences were not significant (χ^2^ = 5.719, *p* = 0.573). Using the Kruskal-Wallis H test followed by Dunn’s post-hoc test with Bonferroni’s correction, we found statistically significant differences in anxiety and depression symptoms (see Table 6 for descriptive statistics and results of between-clusters comparisons). Both in BAI and BDI, Cluster 1 scored significantly lower than Cluster 2 (both *p* < 0.001) and in Cluster 3 (both *p* < 0.001), the differences between Cluster 2 and 3 were not significant.

**Table 6:** BAI, BDI and personality functioning in three clusters (*N* = 149)

	Cluster 1 (*n* = 46)			Cluster 2 (*n* = 37)			Cluster 3 (*n* = 66)			Kruskal-Wallis H test	
	M (SD)	Median	Range	Mean (SD)	Median	Range	Mean (SD)	Median	Range	χ^2^(2)	η^2^
**BAI**	5.07 (4.68)	4	0-18	19.05 (10.96)	16.0	4-44	22.49 (10.64)	20.5	4-52	71.882^*^	0.479
**BDI**	4.22 (4.31)	3	0-22	19.08 (11.6)	16	1-41	26.24 (13.04)	26	0-57	73.671^*^	0.491
**STiP Self**	**0.0 (0.0)**	**0**	**0-0**	**1.0 (0.0)**	**1**	**1-1**	**1.83 (0.6)**	**2**	**1-4**	132.06^*^	**0.891**
**Identity: Boundaries**	0.11 (0.32)	0	0-1	0.73 (0.56)	1	0-2	1.91 (0.91)	2	0-3	89.786^*^	0.601
**Identity: Self-esteem**	0.2 (0.4)	0	0-1	1.41 (0.76)	1	0-3	2.41 (0.91)	2.5	0-4	95.174^*^	0.638
**Identity: Emotions**	0.26 (0.44)	0	0-1	1.24 (0.86)	1	0-3	2.33 (0.85)	2	1-4	90.453^*^	0.606
**Self-direction: Goals**	0.11 (0.32)	0	0-1	0.49 (0.73)	0	0-3	1.68 (1.15)	2	0-4	62.879^*^	0.417
**Self-direction: Norms**	0.04 (0.21)	0	0-1	0.35 (0.72)	0	0-3	1.43 (1.19)	1	0-4	56.882^*^	0.376
**Self-direction: Self-reflection**	0.13 (0.34)	0	0-1	0.95 (0.85)	1	0-3	1.77 (0.87)	2	0-4	74.553^*^	0.497
**STiP Interpersonal**	**0.07 (0.25)**	**0**	**0-1**	**0.65 (0.48)**	**1**	**0-1**	**2.09 (0.78)**	**2**	**1-3**	105.35^*^	**0.708**
**Empathy: Understanding others**	0.13 (0.4)	0	0-2	0.73 (0.87)	1	0-3	1.61 (0.93)	2	0-3	64.732^*^	0.43
**Empathy: Perspectives**	0.2 (0.4)	0	0-1	0.43 (0.56)	0	0-2	1.26 (1.03)	1	0-4	42.515^*^	0.278
**Empathy: Impact**	0.24 (0.52)	0	0-2	0.35 (0.48)	0	0-1	1.38 (0.99)	1	0-3	51.247^*^	0.337
**Intimacy: Connection**	0.11 (0.32)	0	0-1	0.81 (0.91)	1	0-3	1.94 (1.21)	1	0-4	65.731^*^	0.437
**Intimacy: Closeness**	0.3 (0.51)	0	0-2	0.87 (0.86)	1	0-4	2.03 (1.02)	2	0-4	71.73^*^	0.478
**Intimacy: Mutuality**	0.09 (0.29)	0	0-2	0.32 (0.48)	0	0-3	1.48 (1.13)	1	0-3	62.157^*^	0.412
**STiP Total**	**0.02 (0.15)**	**0**	**0-1**	**0.78 (0.42)**	**1**	**0-1**	**2.18 (0.49)**	**2**	**1-3**	129.5^*^	**0.873**

*Note*. * *p* < 0.001. *BAI* Beck Anxiety Inventory, *BDI* Beck Depression Inventory, *STiP* The Semi-structured Interview for Personality Functioning DSM-5

The between-clusters differences in all twelve personality functioning facets were significant with large effect sizes (χ^2^(2) ranging between 42.515 to 95.174, all *p* < 0.001, *η*^2^ between 0.278 and 0.638; see Table 6). Dunn’s post hoc test showed expected between-group differences in the STiP-5.1 total score, Self, and interpersonal domain scores as well as most of the facet scores with Cluster 1 scoring significantly lower than Cluster 2 and Cluster 3, while Cluster 2 showed lower impairment than Cluster 3. However, some statistically non-significant differences were also found. Concretely, the differences between Cluster 1 and Cluster 2 were not significant in these five facets: Self-direction: Goals (*p* = 0.143) and Norms (*p* = 0.306), also in Empathy: Perspectives (*p* = 0.332) and Impact (*p* = 1.0), and Intimacy: Mutuality (*p* = 0.293).

## Discussion

This study investigated the differences in personality functioning in people with personality disorders, with anxiety and mood disorders, and people without psychiatric diagnoses. The data showed significant differences in personality functioning across the groups, indicating the clinical utility of thorough assessment of the facets of personality functioning in a broader spectrum of disorders than only personality disorders for which they were introduced. Furthermore, we used cluster analysis to identify groups based on their personality functioning to delineate those at risk of having personality disorders from those for whom another or no diagnosis is more likely [45]. The cluster analysis delineated three cluster profiles of personality functioning assessed by a semi-structured clinician-rated interview STiP-5.1 [20, 35]. While this supports the relevance of personality functioning assessment in a mental disorders diagnostic process, according to DSM-5 and ICD-11, it also pinpointed some pitfalls, which will be noted below.

Indeed, a simple between-group differences analysis showed expected results with people from the control group scoring significantly lower (all with large effect sizes) in the anxiety and depressive indicators as well as personality functioning. It also was found that, in general, people with personality disorders show higher levels of personality functioning impairment than people with anxiety and mood disorders. Similar to Doering et al. [22], we did not find a significant difference between people with anxiety and mood disorders. Moreover, Doering et al. [22] reported a more severe impairment of personality functioning in people with anxiety disorders comorbid with personality disorders than in those without comorbidity, which aligns with our results clearly showing a higher impairment in people with personality disorders. In addition to their results, we also tested whether people with personality disorders without and with comorbidity differ. In our study, people with personality disorders comorbid with other disorders do not show more severe impairments than those without comorbidity.

The study from Doering et al. [22] is one of the few empirical studies examining personality functioning in other diagnoses than personality disorders. The authors used STIPO [48] for assessment of personality organization. Even though it is not an interview created for assessment of personality functioning implemented in DSM-5 or ICD-11, a large correlation between this instrument and measures of personality functioning according to DSM-5 used in this study was previously found, indicating a convergence of these two models [12, 49] as well as ICD-11 [17]. Doering et al. [22] concluded that anxiety disorders can occur on all levels of personality functioning impairment. However, results of our study are not in line with those from Doering et al. [22], because our results clearly show that people with mood as well as anxiety disorders are found only on the levels 0 to 2 (in total score, as well as Self and Interpersonal), while personality disorders can be found on all levels. Yet fifty people (84.75%) fulfilled level 2 or higher in the total score, thus crossing the Criterion A diagnostic threshold. Level 0 was observed only in two participants in the Interpersonal scale. The scores seem to overlap between the groups, with one group's highest scores being the other group's lowest scores.

These results are further supported by the clustering profiles introduced in this study, which also provide some space for further interpretations. The cluster analysis solution revealed three profiles of personality functioning, with those scoring around 0 being in Cluster 1, those scoring around level 1 belonging to Cluster 2, and those with higher impairment in Cluster 3. These clusters showed 76.51% agreement with the three original study groups and even higher in the groups of healthy controls (81.13%) and personality disorders (87.93%). Therefore, it seems that the overall score as well as two domain scores are a good predictors of the presence of personality disorders, with level 2 impairment as an indicator of a personality disorders diagnosis [50].

On the one hand, these results align with the overlap between the original study groups mentioned before and the principles of clustering analysis, which groups entities that are as similar as possible in one group while also as different as possible from other groups [45]. On the other hand, it can also demonstrate various points connected to personality functioning assessment, such as dimensionality of personality functioning impairments from healthy to personality disturbance, quality of the instrument, differences in diagnostic systems used, and last but not least, the accuracy of clinical judgement both in original diagnostic assessment as well as in the evaluation of the interview used in this study.

Based on our results, it seems that level of personality functioning can serve as an overall indicator of mental health. Particularly the global score and the Self and Interpersonal domain scores, as assessed by the Semi-structured Interview for Personality Functioning DSM-5 (STiP 5.1) [20], seem to delineate well healthy controls from people with personality disorders. Although it seems that the results of this method lack the sensitivity and specificity to distinguish people with other-than-personality-disorders clearly, it points to the dimensionality of anxiety and mood disorders, with some of the patients having more severe personality dysfunctions (some even meeting the personality disorders diagnostic threshold) while others showing only mild impairment of personality functioning. This supports the clinical utility of personality functioning assessment in all people seeking psychiatric help, as it provides valuable information about the severity of the difficulties, for treatment and interventions targeting, predicting ruptures in treatment relationship, or prognosis and clinical outcomes estimation [20, 23].

Furthermore, personality dysfunctions are generally presumed to be long-lasting and relatively stable when compared to some other mental disorders symptoms and syndromes, such as depression or anxiety [1, 5, 31]. Therefore, we also presume that while personality disorders impair personality functioning not only more severely but also more lastingly than mood or anxiety disorders, personality functioning also can share variance with symptom distress and thus be more prone to change over time [51]. This assumption could be supported further by the nature of between-cluster differences in BAI and BDI scores observed in this study. The differences between Cluster 2 and Cluster 3 were not significant, which shows that although one Cluster has significantly lower personality functioning difficulties than the other, anxiety and depressive symptoms were reported at the time of data collection nonetheless. The nature of in-time variability of symptoms and their severity should be addressed in future research.

Nonetheless, as Weekers et al. [52] found out, the diagnostic process according to the DSM-5 alternative model for personality functioning appears to diagnose personality disorders more frequently than previous approaches. We found a similar trend in our study, as more people were assigned to Cluster 3 and met the diagnostic threshold for personality disorders in comparison to the original study groups. It is not clear which model captures personality disorders more accurately. However, it could be suspected that the utility of Criterion A is therefore much broader and could be used as an indicator of the presence of psychopathological syndromes in general [30]. It was even suggested that Criterion A closely aligns with the so-called general factor of psychopathology included in the alternative nosology of mental disorders, the Hierarchical Taxonomy of Psychopathology (HiTOP) [11, 28, 29]. Nonetheless, this could also consequently lead to difficulties with differential assessment between personality disorders and other mental disorders, because many of them can fulfill the diagnostic threshold as well [26]. This can be important especially when using the ICD-11, where the emphasis is primarily on characterizing personality functioning problems, and description using trait domain qualifiers is rather voluntary [5, 53]. Therefore, we concur with Weekers et al. [52] that investigating the continuity, convergence and changes in prevalence of personality disorders is crucial when adapting these new classification systems into clinical practice.

The notion that the personality functioning component could be relevant to psychopathology overall [17, 29] is further elaborated by preliminary findings describing different patterns of associations among domain scores or facets in other-than-personality-disorders diagnoses [21, 24, 25]. The STiP-5.1 total severity score is based on the two domain scores and facet scores rated during the interview; thus, we were interested in differences in these scores as well [26, 27]. In our study, the overall score and two domain scores seem to be an apt indicator of overall mental health. However, looking at between-cluster differences, we can see that some of the twelve facet scores are not showing expected results; concretely, the two facets from the element Self-direction, i.e., Goals and Norms, then Perspective and Impact facets from the element Empathy, and the Mutuality facet that belongs to the Intimacy element. In all these five facets, the differences between Cluster 1 and Cluster 2 were not significant. In light of previous studies, it seems that mood disorders symptoms relate to impairments in self-direction as well as in empathy (especially affective empathy) [54–56]. Mood disorders also relate to the ability for reciprocal cooperation (the Mutuality facet from the Intimacy element) [57]. However, much less is known about the relation of anxiety disorders to these elements. In their study, Clark et al. [57] found depressive but not anxiety and/or stress symptoms related to deficits in a capacity for reciprocal cooperation. Therefore, we think that our results could be explained either by mitigation of these effects by including people with anxiety disorders in our sample or by an insensitivity of some STiP-5.1 facets to more subtle nuances in functioning impairments than others. Furthermore, the scoring is dependent on a clinical judgement, thus we can suspect that for a clinician some facets are easier to capture in a setting of a clinician-rated interview than others, as was for example documented with empathy, which is considered a difficult trait to measure in laboratory as well as in diagnostic processes [58].

Our study has some limitations that should be addressed. First, we should point out the heterogeneity of the patients’ groups, although some of the current research frameworks advise to conduct research on mixed clinical samples to amplify the dimensional and transdiagnostic approach to mental disorders [59, 60]. Notedly, the group of people with mood and anxiety diagnoses was quite heterogeneous in their categorical diagnoses and given the presented findings and paucity of previous studies, we would advise future studies to address these two diagnostic categories separately as well. Unfortunately, we did not verify the diagnoses given to patients by their attending psychiatrists and/or psychologist. However, all patients were recruited in the same institution, where diagnostic procedures are relatively uniform and performed by limited number of people. Additionally, in some rare cases the interviewers were not blind to the clinical status of a participant and their diagnosis, which may have caused some rating bias. Due to organizational and economic reasons we were not able to assess the interrater reliability (ICC); however, all interviewers underwent training led by one of the authors of STiP-5.1, Joost Hutsebaut, regular consensus meetings were held, and good to excellent ICCs were reported in previous studies in various settings and samples (e.g., [15, 20, 36–38]).

We hope that these limitations are outweighed by the strengths of our study, such as the use of the semi-structured clinical interview (STiP-5.1), which is a promising tool for assessment of personality functioning in a broader spectrum of psychopathologies than personality disorders according to AMPD and ICD-11. This is also one of the first studies using the DSM-5-based interview for assessment of personality functioning in other-than-personality-disorders diagnoses, as previous studies (e.g., [17, 22]) applied interviews coming from different theoretical frameworks, i.e., object-relations theory.

## Conclusion

Assessment of the global level of personality functioning impairment constitutes a core of personality psychopathology both in DSM-5 AMPD and ICD-11 [1, 5]. More and more studies indicate that analyzing impairment of personality functioning levels is useful in the clinical assessment of those with other-than-personality disorders as well. In our study, we found that the overall level of personality functioning, as well as the levels of Self and Interpersonal functioning, can be seen as indicators of psychopathological syndromes. The resulting profiles delineating groups based on their personality functioning show that healthy controls were mostly associated with level 0 of personality functioning indicating no impairments, followed by level 1 of personality functioning impairment being frequently associated with mood or anxiety disorders, while, as already set up by diagnostic criteria, moderate or higher levels of impairment (level 2) being indicators of a personality disorder. Furthermore, as the cluster analysis solution indicates, it also could be seen as an indicator of the severity of mental health difficulties and personality disturbances, as well as an indicator of functional abilities. Considering individual impairment in the facets as well, thus could provide valuable information and a broad basis for treatment, therapy, and prognosis.

## Data Availability

The datasets generated and analyzed during the current study are not publicly available due to the sensitive nature of the research and concerns about possible compromise of participants’ privacy, but nonetheless could be available from the corresponding author upon reasonable request.

## Abbreviations

AMPD: Alternative Model for Personality Disorders
BAI: Beck Anxiety Inventory
BDI: Beck Depression Inventory
DSM-5: Diagnostic and Statistical Manual of Mental Disorders – Fifth Edition
HiTOP: Hierarchical Taxonomy of Psychopathology
LPFS: Levels of Personality Functioning Scale
ICD-11: International Classification of Mental and Behavioral Disorders, 11^th^ Edition
PD: Personality Disorder
STiP-5.1 or STiP: The Semi-structured Interview for Personality Functioning DSM-5
STIPO: Structured Interview of Personality Organization
